# When Does a Spin Flip? Arrival Time Distributions and Information Propagation in Discrete Quantum Systems

**DOI:** 10.3390/e28030315

**Published:** 2026-03-11

**Authors:** Lionel Martellini

**Affiliations:** EDHEC Quantum Institute, EDHEC Business School, 06200 Nice, France; lionel.martellini@edhec.edu

**Keywords:** time-of-arrival, quantum measurement, spin systems, Lieb–Robinson bounds, quantum waiting time

## Abstract

We analyze three distinct approaches to time of arrival (TOA) distributions for discrete quantum systems using a spin-$\frac{1}{2}$ particle in a constant magnetic field as a paradigmatic example. We argue that these distributions should not be regarded as competing predictions for the same notion of arrival time, but rather relate to fundamentally different notions whose relevance depends on the physical context. These results are used to analyze information propagation arrival time distributions in XX spin chain systems, and discuss potential applications in quantum information science.

## 1. Introduction

This paper investigates the question of arrival time at a given state in a discrete quantum system. As a paradigmatic example, we ask how long it takes for a spin-$\frac{1}{2}$ particle at rest in a constant magnetic field, initially prepared say in the spin-up state, to be detected in the spin-down state. Clearly, the question “When does a spin flip?” must be understood operationally not in terms of the evolution of an underlying actual state, but in terms of measurement outcomes. In the absence of measurement, the spin undergoes unitary dynamics, evolving into a coherent superposition of up and down states, with the Schrödinger equation deterministically fixing the periodic times at which the transition probability reaches unity. Such times, however, do not correspond to observable events. A spin-flip time can therefore only be defined through measurement statistics, namely as the time at which the target state is first detected within a specified measurement protocol. The present work does not address ontological questions concerning the fundamental nature of time in quantum mechanics, but instead focuses on the operational definition and experimental reconstruction of time arrival distributions within the standard formalism.

If the Born rule provides the probability density for measuring a quantum state at a fixed time, the standard formalism of quantum mechanics, unfortunately, offers no direct prescription for the probability density of a time measurement associated with a fixed state. This asymmetry underlies the so-called time-of-arrival (TOA) problem, which stems from the treatment of time as a parameter rather than an observable and from the absence of a self-adjoint operator canonically conjugate to the Hamiltonian [1], while extensive efforts have been devoted to define arrival-time distributions for continuous systems, with no consensus emerging so far [2,3], comparatively little systematic attention has been devoted to time measurements in discrete quantum systems, especially from a unified operational perspective that explicitly contrasts distinct measurement protocols. Most approaches developed for continuous position measurements do not readily extend to this setting, to the notable exceptions of [4,5], where an application to spin systems is explicitly mentioned, and [6,7] building on earlier work for continuous systems [8,9,10].

Our view is that differences in predicted TOA distributions for both continuous and discrete systems do not merely result from competing approaches to a single notion of TOA, but often reflect divergences or ambiguities in the underlying definition of a quantum arrival time itself. In classical dynamics, a straightforward procedure exists for obtaining an unambiguous notion of arrival time: one may simply continuously monitor the system and record the first hitting time of a target state without altering the motion. In quantum mechanics, however, measurement unavoidably disturbs the system. Repeated projective measurements of a target state collapse the wavefunction and can freeze the dynamics through the quantum Zeno effect [11,12]. Conversely, weak or continuous measurements reduce backaction but lead to non-unitary open-system evolution describable by stochastic master equations or POVMs (positive-operator-valued measures) [13,14]. Consequently, any meaningful definition of a time-of-arrival (TOA) distribution must explicitly specify the measurement protocol under which it is defined.

In this paper, we restrict our attention to the unperturbed unitary evolution of the system and propose a systematic comparison of three operationally distinct notions of arrival time within a single discrete-spin framework. Accordingly, we focus on approaches in which arrival statistics are extracted from single-shot measurements performed at prescribed times, rather than from repeated strong projections or continuous weak monitoring that modify the dynamics. In this context, we extend the existing literature on time-of-arrival (TOA) distributions in discrete quantum systems by examining three distinct notions of TOA for a spin system, analyzing the corresponding probability distributions, and discussing their potential applications. We first show that cumulative time distributions for discrete systems can be inferred from transition probabilities given by the Born rule, which are experimentally accessible through projective measurements performed at discrete times on independently prepared systems. Differentiation of these cumulative distributions yields a first notion of TOA, referred to as the *quantum flow TOA distribution* (or QF distribution for short), closely related to earlier flux and stochastic approach to TOA in [8,9,10], and to the time-of-flow concept introduced operationally in [6,7]. We compare this notion with the quantum clock (QC) approach. The term “quantum clock” is used in different ways in the literature, ranging from physical timekeeping devices employed in quantum metrology to relational constructions in which time is inferred from correlations between subsystems. In the present work, we adopt the operational perspective developed in [4,5], where a clock degree of freedom is introduced and time is defined conditionally from joint measurements on the system and the clock. Within this framework, one asks: given that the system is detected in a specified arrival state, what is the probability distribution of the corresponding clock reading? The resulting time distribution is therefore conditional upon successful detection of the target state, and differs conceptually from flow-based or first-detection constructions. We emphasize that our use of the quantum clock approach serves as a benchmark notion of arrival time within a unified operational comparison, rather than as the central focus of the present analysis. We finally introduce another notion, termed the *quantum waiting time distribution* (or QW distribution for short), defined as the waiting time distribution until a first detection of the target state occurs under single measurements on an ensemble of identical systems. Since each measurement is performed on an independent copy of the system, the resulting QW TOA distribution characterizes the stopping time of an experimental protocol rather than a fundamental property of the system’s dynamics itself such as the first-arrival time of a single system undergoing successive strong measurements. As such, the QW TOA distribution represents a protocol-dependent observable capturing the latency of a measurement procedure. We also provide an explicit experimental protocol for the empirical reconstruction of each distribution via discrete-time projective measurements, in the spirit of stroboscopic measurement schemes discussed in [6,7,8,9,10,15]. We use these results to analyze information propagation time distributions in XX spin chain systems via the quantum wait TOA distribution and analyze its relation with the Lieb–Robinson bound [16]. Finally, we conclude by emphasizing that the operational relevance of each notion of arrival time is context dependent, and that each approach naturally gives rise to distinct applications in quantum sensing, computing, and communication.

## 2. Transition Probabilities and Quantum Flow TOA Distribution for a 2-State Spin System

For concreteness, we cast the bulk of our discussion in a simple 2-state model, but our results can be readily extended to more general settings, including closed or open *N*-state systems, as discussed in a later section. Specifically, let us first consider the Hamiltonian for an electron (or any spin-$\frac{1}{2}$ particle) at rest in a magnetic field of constant magnitude oriented along the x-axis $H=\frac{\hbar \omega_{0}}{2}\sigma_{x}$. The Schrödinger equation of motion for the spin state $|\psi_{t}\rangle$ reads

(1) $$H|\psi_{t}\rangle =i\hbar \frac{d}{dt}|\psi_{t}\rangle ,$$

which solution given the initial state $|\psi_{0}\rangle$ is

(2) $$|\psi_{t}\rangle =e^{-\frac{i}{\hbar }Ht}|\psi_{0}\rangle ,$$

with

(3) $$\begin{array}{ccc} e^{-\frac{i}{\hbar }Ht} & = & \cos\left(\frac{\omega_{0}t}{2}\right)I-i\sin\left(\frac{\omega_{0}t}{2}\right)\sigma_{x}. \end{array}$$

### 2.1. Transition Probabilities for a 2-State Spin System

Starting from a general superposed state $|\psi_{0}\rangle =cos\theta |0\rangle +e^{i\varphi }\sin\theta |1\rangle$, we obtain the probabilities of measuring the up state |0〉 and the down state |1〉 at time *t* as follows:

(4) $$p_{u}\left(t\right)=\cos^{2}\theta \cos^{2}\left(\frac{\omega_{0}t}{2}\right)+\sin^{2}\theta \sin^{2}\left(\frac{\omega_{0}t}{2}\right)+I\left(t\right),$$

and

(5) $$p_{d}\left(t\right)=\sin^{2}\theta \cos^{2}\left(\frac{\omega_{0}t}{2}\right)+\cos^{2}\theta \sin^{2}\left(\frac{\omega_{0}t}{2}\right)-I\left(t\right),$$

where I(t) represents an interference term given by

(6) $$I\left(t\right)=-2\cos\theta \sin\theta \sin\varphi \cos\left(\frac{\omega_{0}t}{2}\right)\sin\left(\frac{\omega_{0}t}{2}\right).$$

Noting that $p_{u}\left(0\right)=\cos^{2}\theta$ and $p_{d}\left(0\right)=\sin^{2}\theta$, we can rewrite the time *t* probabilities as linear functions of the time 0 probabilities up to the interference term:

(7) $$\begin{array}{ccc} p_{u}\left(t\right) & = & p_{u}\left(0\right)p_{u\to u}\left(t\right)+p_{d}\left(0\right)p_{d\to u}\left(t\right)+I\left(t\right), \end{array}$$

(8) $$\begin{array}{ccc} p_{d}\left(t\right) & = & p_{d}\left(0\right)p_{d\to d}\left(t\right)+p_{u}\left(0\right)p_{u\to d}\left(t\right)-I\left(t\right). \end{array}$$

Here, the *transition probability* $p_{i\to j}\left(t\right)$ (for i,j=u,d) can be interpreted as the conditional probability of measuring the system in the state *j* at time *t* given that it was prepared in the state *i* at time 0

(9) $$p_{i\to j}\left(t\right)={\left|\langle j|U(t)|i\rangle \right|}^{2},$$

where we have $p_{u\to u}\left(t\right)=p_{d\to d}\left(t\right)=\cos^{2}\left(\frac{\omega_{0}t}{2}\right)$ and $p_{u\to d}\left(t\right)=p_{d\to u}\left(t\right)=\sin^{2}\left(\frac{\omega_{0}t}{2}\right)$. The interpretation of these transition probabilities is straightforward. For example, $p_{u\to u}\left(t\right)$ represents the probability that a system prepared in the up state at time 0 is still measured in the up state at time *t*. This quantity, which is known as the *survival probability*, is also referred to as the *fidelity* between the initial state the state at time *t*. On the other hand, $p_{u\to d}\left(t\right)$ is the probability that the system has transitioned to the down state by time *t*.

### 2.2. Quantum Flow TOA Distribution for a 2-State Spin System

In survival analysis, transition probabilities are typically used to obtain the cumulative distribution of the time-of-arrival at a given state. Our approach to introducing a relevant notion of quantum TOA exploits this feature by noting that the proportion of particles that have left the *up* state to arrive at the *down* state between time 0 and time *t*, which we denote by $\Pi_{u\to d}^{QF}\left(t\right)$ or simply $\Pi_{d}^{QF}\left(t\right)$, is directly given by $p_{u}\left(0\right)p_{u\to d}\left(t\right)$.

**Remark** **1.** *One may alternatively define arrival in terms of the full occupation probability $p_{d}\left(t\right)$, thereby incorporating both the interference contribution I(t) and the component corresponding to systems that were initially in the down state and remain there. Such a definition would therefore not isolate the population flow from the initially prepared up state, but would instead mix coherent superposition effects, initial occupation of the down state, and genuine transition dynamics. In the present work, we adopt the stricter flow-based definition, which captures only transitions from up to down.*

Using $\Pi_{d}^{QF}\left(t\right)\equiv \int_{0}^{t}\pi_{d}^{QF}\left(s\right)\;ds$, where $\pi_{d}^{QF}\left(t\right)$ is the probability *density* function for the quantum flow TOA, denoted by $\tau_{d}^{QF}$, at the down state, we obtain by differentiating Equation (8):

(10) $$\Pi_{d}^{QF}\left(t\right)\equiv \mathrm{Pr}\left(\tau_{d}^{QF}\leq t\right)=p_{u}\left(0\right)p_{u\to d}\left(t\right).$$

Since $\Pi_{d}^{QF}\left(t\right)=\int_{0}^{t}\pi_{d}^{QF}\left(s\right)ds$, the probability density for the time-of-arrival (TOA) at the down state can be obtained by differentiating Equation (10) as

(11) $$\pi_{d}^{QF}\left(t\right)=\frac{d}{dt}\Pi_{d}^{QF}\left(t\right)=p_{u}\left(0\right)\frac{d}{dt}p_{u\to d}\left(t\right),$$

or equivalently from (8):

(12) $$\pi_{d}^{QF}\left(t\right)=\frac{d}{dt}p_{d}\left(t\right)-p_{d}\left(0\right)\frac{d}{dt}p_{d\to d}\left(t\right)+\frac{d}{dt}I\left(t\right).$$

**Remark** **2.** *If the time-derivative of the conditional probability $\frac{d}{dt}p_{u\to d}\left(t\right)$ in Equation (11) is negative, then the corresponding outflow of probability should be interpreted as a time-of-departure distribution. For example, the time derivative of the down-state occupation probability for the standard spin system becomes negative on $\left[\frac{\pi }{\omega_{0}},\frac{2\pi }{\omega_{0}}\right]$. This is because the system is periodic and involves oscillatory occupation, making the transition from the up state to the down state effectively reversible. Moreover, an appropriate normalization must be applied to ensure that the integral of the corresponding TOA distribution equals 1, as is mentioned in the application to the standard spin system.*

For our 2-state spin model, we have

(13) $$\pi_{d}^{QF}\left(t\right)=\cos^{2}\theta \;\frac{d}{dt}\sin^{2}\left(\frac{\omega_{0}t}{2}\right)=\frac{1}{2}\omega_{0}\cos^{2}\theta \;\sin\left(\omega_{0}t\right).$$

Since the system is periodic with a first down measurement that will occur with probability 1 within the interval $\left[0,\frac{\pi }{\omega_{0}}\right]$, we may restrict our attention to this interval and obtain

(14) $$\int_{0}^{\frac{\pi }{\omega_{0}}}\pi_{d}^{QF}\left(t\right)\;dt=\cos^{2}\theta ,$$

which coincides with the probability of measuring an up state at the initial time. That the integral of the distribution is not equal to 1 but to $p_{u}\left(0\right)$ is consistent with the fact that it is only when the initial state is up that an arrival to the down state can occur. In other words, we can define the *normalized* distribution

(15) $${\tilde{\pi }}_{d}^{QF}\left(t\right)=\frac{1}{2}\omega_{0}\sin\left(\omega_{0}t\right)I_{\left[0,\frac{\pi }{\omega_{0}}\right]}\left(t\right),$$

represents the distribution of the time-of-arrival at the down state *conditional upon an arrival taking place*.

**Remark** **3.** *In the specific non-superposed case of a system that is initially prepared in the down state with probability 1 (i.e., when $\theta =\frac{\pi }{2}$), we have $p_{u}\left(0\right)=0$ and I(t)=0. In this situation, from (12) we determine that the TOA distribution is given by the time derivative of the time t probability for the system to be measured in the up state*

(16) $$\pi_{u}^{a}\left(t\right)=\frac{d}{dt}p_{u}\left(t\right),$$

*where $p_{u}\left(t\right)=\sin^{2}\left(\frac{\omega_{0}t}{2}\right)$. This result is consistent with the findings in [6], but we emphasize that it is only in the specific case of an initial non-superposed state that the TOA distribution coincides with the time-derivative of the occupation probability, which is called time-of-flow (or TF in short) distribution in [6]. For a general superposed state, our predicted TOA distribution is different from the TF distribution $\frac{d}{dt}p_{u}\left(t\right)$, since Equation (12) contains additional terms that are non-zero in general.*

**Figure 1:**
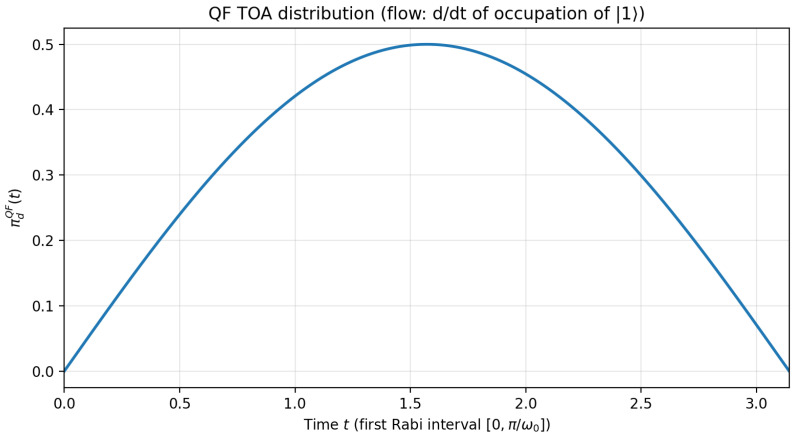
Predicted distribution for the QF arrival time. This figure shows the predicted arrival time distribution $\pi_{d}^{QF}\left(t\right)$ at the down state (Equation (13)) on the time interval $\left[0,\frac{\pi }{\omega_{0}}\right]$. For this figure, we set $\omega_{0}=1$ (equivalently, we measure time in units of $1/\omega_{0}$, i.e., $t^{\prime }=\omega_{0}t$).

For illustration and comparison with other notions of TOA and their associated distribution, Figure 1 shows the theoretical prediction $\pi_{d}^{QF}\left(t\right)$ for the TOA at the down state assuming $p_{u}\left(0\right)=1$. The distribution in figure first increases until the midpoint of the interval, and then starts decreasing, reflecting the fact that a first up state measurement then becomes increasingly likely to have been obtained before, where “before” relates to independent measurement on identical systems and not repeated projective measurements on the same system, which would induce repeated state collapse and an associated Zeno effect. In the limit, when approaching $t=\frac{\pi }{\omega_{0}}$, it becomes extremely likely that an up-state outcome could have been obtained if an earlier measurement had been performed.

### 2.3. Experimental Protocol Associated with Quantum Flow TOA Distributions

As recalled in the introduction, one key challenge in the literature about time-of-arrival is the presence of divergences, or even worse ambiguities, in the underlying definition of a time measurement. The most efficient way to resolve ambiguities related to the abuses of language involved in such definitions is to describe as precisely as possible the experimental procedure that is associated with the observable for which we are deriving the distribution. In what follows, we precisely present an experimental protocol that can be used to generate the empirical distribution ${\hat{\pi }}_{d}\left(t\right)$ associated with the observables that we define as time-of-arrival at the down state. We emphasize that we focus on single measurement protocols because performing repeated measurements would result in setting the system in the newly measured state, and would thus amount to restarting the analysis from fresh on each measurement date. This series of single measurements are called “stroboscopic measurements” in [15] (see also [6,7,8] for an application to TOA distributions in discrete and continuous systems, respectively).

We see from Equation (11) that the TOA distribution involves the product of two terms, $p_{u}\left(0\right)$ and $\frac{d}{dt}p_{u\to d}\left(t\right)$. The first term, $p_{u}\left(0\right)$, is directly obtained from the initial preparation of the system, and it is therefore the second term that requires attention. The experimental protocol that can be used to estimate $\frac{d}{dt}p_{u\to d}\left(t\right)$ is as follows: We split the interval $\left[0,\frac{\pi }{\omega_{0}}\right]$ into *K* equally spaced time increments kδt, where δt is the temporal resolution, with $K\delta t=\frac{\pi }{\omega_{0}}$, and then perform the following step-by-step procedure: (1) since we are interested in the transition from the up state to the down state, one initially (at $t_{0}=0$) prepares a spin system in the up state |0〉 using a standard Stern–Gerlach device; (2) one makes a spin measurement at some later time $t_{k}=k\delta t$; (3) one repeats this procedure a large number *N* of times while performing the spin measurement at the exact same time $t_{k}=k\delta t$ and record the number $N_{k\delta t}^{u\to d}$ of down state |1〉 spin measurements at that time starting from an initial up state; (4) one repeats the steps (1) to (3) for each time $t_{k}=k\delta t$ with k=1,…,K.

From this protocol, one may compute for all dates $t_{k}$ the sample statistic corresponding to the probability distribution of a transition from an up to a down spin measurement

(17) $${\hat{p}}_{u\to d}\left(t_{k}\right)\equiv \frac{N_{k\delta t}^{u\to d}}{N}\underset{N\to \infty }{⟶}p_{u\to d}\left(t_{k}\right)=\sin^{2}\frac{\omega_{0}t_{k}}{2}.$$

The statistic corresponding to the TOA distribution $\pi_{d}\left(t_{k}\right)$ can then be obtained by multiplying $\frac{1}{\delta t}\frac{N_{k\delta t}^{u\to d}-N_{(k-1)\delta t}^{u\to d}}{N}$ by the initial probability $p_{u}\left(0\right)$ of an up state for the prepared system:

(18) $$\begin{array}{cc} {\hat{\pi }}_{d}^{QF}\left(t_{k}\right) & =p_{u}\left(0\right)\frac{1}{\delta t}\frac{N_{k\delta t}^{u\to d}-N_{(k-1)\delta t}^{u\to d}}{N} \end{array}$$

(19) $$\begin{array}{cc} \; & \underset{N\to \infty }{⟶}p_{u}\left(0\right)\frac{1}{\delta t}\left(p_{u\to d}\left(t_{k}\right)-p_{u\to d}\left(t_{k}-\delta t\right)\right) \end{array}$$

(20) $$\begin{array}{cc} \; & \underset{\delta t\to 0}{⟶}p_{u}\left(0\right)\frac{d}{dt}p_{u\to d}\left(t_{k}\right)=\pi_{d}^{QF}\left(t_{k}\right). \end{array}$$

The analysis can easily be transposed to a focus on the up state instead of the down state, or a focus on time-of-departure as opposed to TOA distributions. For example, a similar experimental protocol can be used to estimate the transition probability $p_{d\to u}\left(t\right)$, which is needed for the empirical estimation of the TOD distribution from the down state, based on the number of particles $N_{k\delta t}^{d\to u}$ measured in the up state starting from an initial down state preparation. For this system, the transition matrix is symmetric with $p_{d\to u}\left(t\right)=p_{u\to d}\left(t\right)$, so we need not repeat the procedure in this case.

### 2.4. Extension to General N-State Discrete Quantum Systems

These results extend in a straightforward manner to any general *N*-state system by defining the transition probability from an initial state |i〉 at date 0 to a final state |j〉 at time *t* as

(21) $$p_{i\to j}\left(t\right)={\left|\langle j|U(t)|i\rangle \right|}^{2}.$$

Starting from a general superposed state $|\psi_{0}\rangle =\sum_{i}c_{i}|i\rangle$ satisfying standard normalization conditions, the corresponding time-evolved state is $|\psi_{t}\rangle =U\left(t\right)|\psi_{0}\rangle =\sum_{i}c_{i}U\left(t\right)|i\rangle$. Hence,

(22) $$\begin{array}{c} p_{j}\left(t\right)={\left|\langle j|\psi_{t}\rangle \right|}^{2}=\sum_{i}|c_{i}{|}^{2}{\left|\langle j|U(t)|i\rangle \right|}^{2}+\sum_{i\neq i^{\prime }}c_{i}c_{i^{\prime }}^{*}\langle j|U(t)|i\rangle \langle i^{\prime }\left|U{\left(t\right)}^{†}\right|j\rangle , \end{array}$$

where the second term on the right-hand side is an interference term that we denote by $I_{j}\left(t\right)$. As a result, we obtain the following decomposition

(23) $$\begin{array}{c} p_{j}\left(t\right)=p_{j}\left(0\right)p_{j\to j}\left(t\right)+\sum_{i\neq j}p_{i}\left(0\right)p_{i\to j}\left(t\right)+I_{j}\left(t\right). \end{array}$$

To derive the cumulative probability distribution of the arrival time at the |j〉 state between time 0 and time *t*, denoted by $\Pi_{j}^{QF}\left(t\right)\equiv \int_{0}^{t}\pi_{j}^{QF}\left(s\right)ds$, we note again that it is *by definition* given by the total proportion of particles that have arrived at the |j〉 state over the period. Summing up over all possible departure states, the proportion of particles that have arrived at state |j〉 between the initial time and time *t* is given by

(24) $$\Pi_{j}^{QF}\left(t\right)\equiv \mathrm{Pr}\left(\tau_{j}^{QF}\leq t\right)=\sum_{i\neq j}p_{i}\left(0\right)p_{i\to j}\left(t\right),$$

and the arrival time distribution at state |j〉 is therefore

(25) $$\pi_{j}^{QF}\left(t\right)=\frac{d}{dt}\Pi_{j}^{QF}\left(t\right)=\sum_{i\neq j}p_{i}\left(0\right)\frac{d}{dt}p_{i\to j}\left(t\right),$$

which generalizes Equation (11) to multi-state systems.

The corresponding experiment protocol would be as follows: We first prepare at the initial time a large number *N* of independent systems in the |i〉 state and record the proportion measured in the |j〉 at date $t_{k}$. We then repeat this protocol for each one of the N(N−1) of the transition probabilities $p_{i\to j}\left(t_{k}\right)$, and each one of the possible dates $t_{k}$ for k=1,…,K. For time reversal invariant systems, the transition matrix is symmetric (that is, $p_{i\to j}\left(t\right)=p_{j\to i}\left(t\right)$) so the total number of transition probabilities to estimate is divided by a factor of 2 and reduces to $\frac{N(N-1)}{2}$. Additionally, one needs to use the usual procedure to estimate the probabilities for each given state both at the initial time ($p_{i}\left(0\right)$ for i=1,…,M) and at time $t_{k}$ ($p_{i}\left(t_{k}\right)$ for i=1,…,M and k=1,…,K).

The analysis directly encompasses the case of mixed initial states. To see this, let ${\left\{|i\rangle \right\}}_{i=1}^{N}$ be an orthonormal basis and $\Pi_{i}\equiv |i\rangle \;\langle i|$. Consider a general mixed initial state

(26) $$\rho_{0}=\sum_{i,i^{\prime }=1}^{N}\rho_{ii^{\prime }}\;|i\rangle \;\langle i^{\prime }|,\;\rho_{0}\geq 0,\;\mathrm{Tr}\left(\rho_{0}\right)=1.$$

We define the initial occupation of |i〉 by

(27) $$p_{i}\left(0\right)\equiv \mathrm{Tr}\left(\Pi_{i}\rho_{0}\right)=\rho_{ii}.$$

For $U\left(t\right)=e^{-iHt}$ and $\rho \left(t\right)=U\left(t\right)\rho_{0}U^{†}\left(t\right)$, define the transition probability

(28) $$p_{i\to j}\left(t\right)\equiv \mathrm{Tr}\;\left[\Pi_{j}\;U\left(t\right)\Pi_{i}U^{†}\left(t\right)\right]=|\langle j|U(t)|i\rangle {|}^{2}.$$

The QF cumulative arrival probability at |j〉 by time *t* is defined as the total proportion of the population that has arrived at |j〉 from any other basis state,

(29) $$\Pi_{j}^{QF}\left(t\right)=\sum_{i\neq j}\mathrm{Tr}\left(\Pi_{i}\rho_{0}\right)\;\mathrm{Tr}\;\left[\Pi_{j}\;U\left(t\right)\Pi_{i}U^{†}\left(t\right)\right].$$

and differentiating yields the QF TOA density.

Finally, the analysis can also be extended to open systems interacting with their environment. For this, let the reduced evolution be a CPTP map $\Phi_{t}$, so that $\rho \left(t\right)=\Phi_{t}\left(\rho_{0}\right)$. A natural generalization of the basis transition probability is

(30) $$p_{i\to j}\left(t\right)\equiv \mathrm{Tr}\;\left[\Pi_{j}\;\Phi_{t}\left(\Pi_{i}\right)\right].$$

The corresponding QF cumulative arrival probability and density are defined by the same mixture-over-departure-states construction:

(31) $$\begin{array}{cc} \Pi_{j}^{QF}\left(t\right)\; & =\sum_{i\neq j}p_{i}\left(0\right)\;p_{i\to j}\left(t\right)=\sum_{i\neq j}\mathrm{Tr}\left(\Pi_{i}\rho_{0}\right)\;\mathrm{Tr}\;\left[\Pi_{j}\;\Phi_{t}\left(\Pi_{i}\right)\right], \end{array}$$

(32) $$\begin{array}{cc} \pi_{j}^{QF}\left(t\right)\; & =\frac{d}{dt}\Pi_{j}^{QF}\left(t\right)=\sum_{i\neq j}\mathrm{Tr}\left(\Pi_{i}\rho_{0}\right)\;\frac{d}{dt}\mathrm{Tr}\;\left[\Pi_{j}\;\Phi_{t}\left(\Pi_{i}\right)\right]. \end{array}$$

If $\Phi_{t}=e^{tL}$ is Markovian with Lindbladian generator L, then $p_{i\to j}\left(t\right)=\mathrm{Tr}\;\left[\Pi_{j}\;e^{tL}\left(\Pi_{i}\right)\right]$ and

(33) $$\pi_{j}^{QF}\left(t\right)=\sum_{i\neq j}\mathrm{Tr}\left(\Pi_{i}\rho_{0}\right)\;\frac{d}{dt}\mathrm{Tr}\;\left[\Pi_{j}\;e^{tL}\left(\Pi_{i}\right)\right].$$

## 3. Comparison with Other Notions of TOA

As recalled in the introduction, there exists another notion of arrival time for a discrete system, which has been referred to as the *quantum clock* (QC) approach [4,5], and which addresses a different question, namely: *given that the system has been measured in the arrival state (say the down state for the spin system), what is the probability that a clock at the lab reads time t?*

### 3.1. Quantum Clock (QC) Arrival Time Distribution

This approach shares a common feature with the QF approach in that the predicted TOA distribution can also be derived from state occupation probabilities inferred by the Born rule. As a result, the experimental construction of the QC TOA distribution can be derived from occupation state frequencies extracted from the same (single-measurement/stroboscopic) protocol that can be used to obtain empirical distributions for the QF TOA notion.

More specifically, assume for simplicity that the system is prepared in the up state |0〉, that is assume $|\psi_{0}\rangle =|0\rangle$. The QC prediction is that the TOA distribution at the down state is proportional to the probability of measuring a down state at time *t*

(34) $$\pi_{d}^{QC}\left(t\right)\propto p_{d}\left(t\right)=\sin^{2}\left(\frac{\omega_{0}t}{2}\right),$$

which differs from the QF TOA distribution that is instead proportional to the time derivative $\frac{d}{dt}p_{u\to d}\left(t\right)$.

The experimental protocol used to extract the quantum clock TOA distribution is the exact same as the one presented before, except for the last step. We first split the interval $\left[0,\frac{\pi }{\omega_{0}}\right]$ into *K* equally spaced time increments kδt. At $t_{0}=0$, we prepare a spin system in the up state |0〉. We then make a spin measurement at some later time $t_{k}=k\delta t$, and repeat *N* times and record number $N_{k\delta t}^{u\to d}$ of down state measurements. Subsequently, we repeat the previous steps for each time $t_{k}=k\delta t$ with k=1,…,K. Then, as a final step, we compute the statistic for TOA *by* time *t*

(35) $$\begin{array}{cc} {\hat{\pi }}_{d}^{QC}\left(t_{k}\right) & =\frac{1}{\delta t}\frac{N_{k\delta t}^{u\to d}}{\sum_{k^{\prime }=1}^{K}N_{k^{\prime }\delta t}^{u\to d}} \end{array}$$

(36) $$\begin{array}{cc} \; & \underset{\begin{array}{c} N\to \infty \\ \delta t\to 0 \end{array}}{⟶}\pi_{d}^{QC}\left(t_{k}\right). \end{array}$$

**Figure 2:**
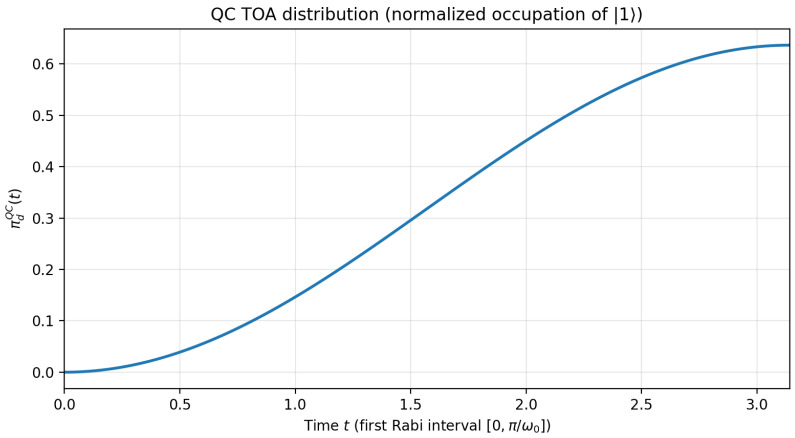
Predicted distribution for the QC arrival time. This figure shows the predicted waiting time distribution $\pi_{d}^{QC}\left(t\right)$ until a first down state is measured (Equation (34)) on the time interval $\left[0,\frac{\pi }{\omega_{0}}\right]$. For this figure, we set $\omega_{0}=1$ (equivalently we measure time in units of $1/\omega_{0}$, i.e., $t^{\prime }=\omega_{0}t$).

In Figure 2, we report the quantum clock prediction $\pi_{d}^{QC}\left(t\right)$. In contrast with the QF and QW distributions, the QC distribution is strictly increasing on the interval $\left[0,\frac{\pi }{\omega_{0}}\right]$, which reflects the fact that the probability of a down state measurement keeps increasing on this interval. Again such differences are consistent with underlying differences in the associated notion of arrival time.

### 3.2. Quantum Waiting Time (QW) Distribution

While the quantum clock (QC) distribution represents a natural description of the conditional probability of a time measurement given a specific state measurement, it does not capture the notion of a first observed transition. We now turn to a distinct time-of-arrival concept, which we refer to as *quantum waiting time* (or QW distribution for short), and which addresses yet another question, namely: *how long must one wait before one system in an ensemble is observed in the arrival state for the first time?*

For concreteness, we present the concept in the 2-state spin system. We are specifically interested in the probability that the first down spin measurement of a set of *N* identical particles happened at time *t*. For this, we consider again a small time interval and we construct the *survival probability* that a succession of single spin measurements performed on a large number of identical particles has generated only up spin outcomes before that date:

(37) $$S\left(t_{k}\right)=\prod_{k/k\delta t<t}\left(1-p_{d}\left(t_{k}\right)\right)=\prod_{k/k\delta t<t}\left(1-\sin^{2}\left(\frac{\omega_{0}t_{k}}{2}\right)\right)$$

where $t_{k}=k\delta t$. The probability that a first down measurement on identically prepared particles is exactly obtained at date $t_{k}$ is then

(38) $${\hat{\pi }}_{d}^{QW}\left(t_{k}\right)=\left[\prod_{k/k\delta t<t}(1-p_{d}\left(k\delta t\right)\right]p_{d}\left(t_{k}\right).$$

Unlike continuous-time detection models based on a hazard rate formulation, in which a time-dependent detection rate λ(t) is introduced and the first-arrival distribution is written as $\pi \left(t\right)=\lambda \left(t\right)\exp\;\left(-\int_{0}^{t}\lambda \left(s\right)\;ds\right)$ (see, e.g., [17,18]), the present QW distribution is derived strictly from a discrete stroboscopic protocol in which a single projective measurement is performed at each sampling time on independent identically prepared systems. The distribution therefore depends on the temporal resolution δt, which represents an experimental parameter.

**Figure 3:**
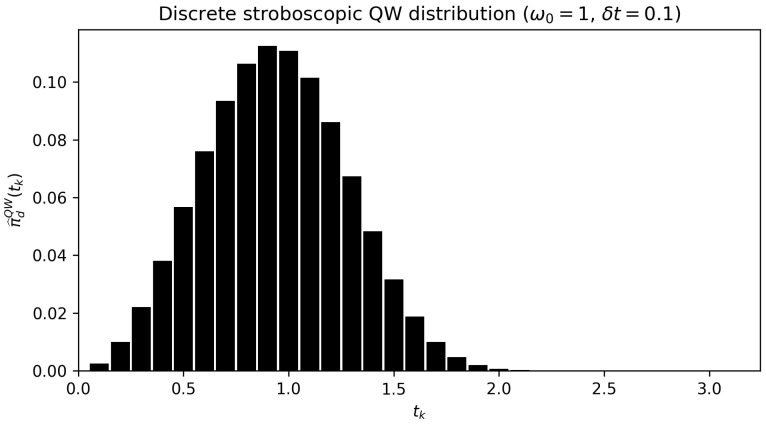
Discrete stroboscopic quantum waiting time distribution. Histogram representation of the empirical first-detection distribution ${\hat{\pi }}_{d}^{QW}\left(t_{k}\right)$ obtained under the single-shot stroboscopic protocol with sampling times $t_{k}=k\;\delta t$, temporal resolution δt=0.1, and $\omega_{0}=1$. The theoretical prediction is ${\hat{\pi }}_{d}^{QW}\left(t_{k}\right)=S_{k}\;p_{d}\left(t_{k}\right)$, where $S_{k}=\prod_{j<k}\left(1-p_{d}\left(t_{j}\right)\right)$. No continuous-time hazard approximation is invoked.

Figure 3 shows the theoretical prediction $\pi_{d}^{QW}\left(t\right)$ for the TOA at the down state assuming $p_{u}\left(0\right)=1$ and δt=0.1. For sufficiently small δt, the histogram–density $\mathrm{Pr}(T=t_{k})/\delta t$ captures the buildup and subsequent decay of first-detection probability. The peak of the distribution reflects the ballistic timescale set by the system dynamics, while the early time suppression follows from the small Born probability $p_{d}\left(t_{k}\right)$ and the discrete survival product $S_{k}$. Importantly, no continuous monitoring or hazard-rate assumption is invoked.

We now turn to an empirical procedure that allows us to construct the histogram associated with this quantum waiting time (QW) predicted distribution. We split again the $\left[0,\frac{\pi }{\omega_{0}}\right]$ interval into *K* equally spaced time increments kδt, where δt is the temporal resolution and implement the following step-by-step procedure. (1) One initially (at time $t_{0}=0$) prepare *K* identical spin systems in the |0〉 state. (2) For the first particle one performs a spin measurement at time $t_{1}=\delta t$, for the second particle one performs a spin measurement at time $t_{2}=2\delta t$, for the third particle one performs a spin measurement at time $t_{3}=3\delta t$, …, and one goes on until one obtains a first |1〉 spin measurement at some time $t_{k}$ that is recorded. (3) One repeats steps (1) and (2) a very large number *N* of times, and one denotes by $M_{k\delta t}$ the number of times when the outcome of the procedure yields the waiting time $t_{k}$ for the first down state measurement. This procedure allows one to obtain the following empirical probability distribution of the time measurements as

(39) $${\hat{\pi }}_{d}^{QW}\left(t_{k}\right)=\frac{M_{k\delta t}}{N}$$

since $\sum_{k^{\prime }=1}^{K}M_{k\delta t^{\prime }}=N$. It should be noted that this statistic can alternatively be computed as follows:

(40) $${\hat{\pi }}_{d}^{QW}\left(t_{k}\right)=\prod_{k^{\prime }=1}^{k}\left(1-\frac{N_{k^{\prime }\delta t}}{N}\right),$$

which shows that it can be extracted on the basis of the same exact experimental protocol as the statistics associated with the QF distribution. In other words, we are using the same protocol to compute two different statistics that correspond to two different observables, and it is up to the experimenter to decide which quantity is of the highest degree of interest depending on the physical question at hand.

## 4. Information Propagation Time Arrival Distributions in XX Spin Chain Systems

In this section, we apply these results to a simple one-dimensional XX spin chain model of *L* qubits with nearest-neighbor couplings and with a Hamiltonian given by

(41) $$H\;=\;\frac{J}{2}\sum_{n=1}^{L-1}\left(\sigma_{n}^{x}\sigma_{n+1}^{x}+\sigma_{n}^{y}\sigma_{n+1}^{y}\right),\;J>0,$$

where *J* denotes the nearest-neighbor coupling strength. This XX spin chain provides a simple analytically tractable toy model where coherent excitation transport gives rise to information propagation. At t=0, starting from the fully polarized state with all spins down, let us assume one applies a flip $U_{0}=X_{0}$, creating a single spin-up excitation which propagates along the chain over time, and let

|n〉≡|0⋯010⋯0〉

denote the basis state with a single spin-up excitation at site *n* and all other spins down.

In this model and starting from a fully polarized state, a single local spin flip confines the dynamics to the one-excitation subspace spanned by {|n〉}. The XX Hamiltonian maps via the Jordan–Wigner transformation to a nearest-neighbor tight-binding model of free fermions [19], and the transition amplitude to propagate from site *a* to site *b* at time *t* can therefore be written in closed form as

$$\langle b|e^{-iHt}|a\rangle =i^{d}J_{d}\left(2Jt\right),\;d=\left|b-a\right|,$$

so that the corresponding (unnormalized) detection probability takes the form

(42) $$p_{\det}\left(t\right)\;\propto \;{\left[J_{d}\left(\alpha Jt\right)\right]}^{2},\;d=\left|b-a\right|,$$

where $J_{d}$ denotes the Bessel function of the first kind and α=2 for the normalization used here. From Equation (42), it can be seen that for times t≪d/(αJ), the detection probability is exponentially small in *d*, implying a strong suppression of early arrivals. Furthermore, the probability mass concentrates around a *typical arrival time*

(43) $$t_{\mathrm{typ}}∼\frac{d}{\alpha J},$$

which scales linearly with the distance between source and target. This model can also be shown to give rise to a light-cone structure in information propagation through the Lieb–Robinson (LR) bound [16,20]. More precisely, consider two local observables $A_{a}$ and $B_{b}$ supported, respectively, at sites *a* and *b*, with lattice distance d=|b−a|. Denoting Heisenberg evolution by

$$A_{a}\left(t\right)=e^{iHt}A_{a}e^{-iHt},$$

the LR bound states that there exist constants C,μ>0 such that

(44) $$∥\left[A_{a}\left(t\right),B_{b}\right]∥\;\leq \;C\;∥A_{a}∥\;∥B_{b}∥\;\exp\;\left[-\mu \left(d-v_{\mathrm{LR}}\;\left|t\right|\right)\right],$$

where $v_{\mathrm{LR}}$ is the Lieb–Robinson velocity, which scales linearly with the interaction strength *J* for finite-range Hamiltonians. The LR velocity $v_{\mathrm{LR}}$ provides an upper bound on information propagation in the sense of exponential suppression of correlations, and hence of operational influence, outside the cone.

We now complement the Lieb–Robinson bound with a distribution of information arrival times defined operationally under the single-shot quantum wait stroboscopic protocol introduced earlier, which is well suited for analyzing arrival time distributions in latency protocols. For this, we consider sampling times $t_{k}=k\;\delta t$ on a set of independent, identically prepared copies of the system. At each time $t_{k}$, a projective measurement is performed at site *b* to test for the presence of the excitation. The point detection probability at time $t_{k}$ is

(45) $$p_{\det}\left(t_{k}\right)=\left|\langle b\right|e^{-iHt_{k}}|a\rangle {|}^{2}=J_{d}{\left(2Jt_{k}\right)}^{2},$$

where d=|b−a|. The first-detection probability mass function is then

(46) $${\hat{\pi }}_{d}^{QW}\left(t_{k}\right)=\left(\prod_{j=1}^{k-1}\left(1-p_{\det}\left(t_{j}\right)\right)\right)p_{\det}\left(t_{k}\right).$$

**Figure 4:**
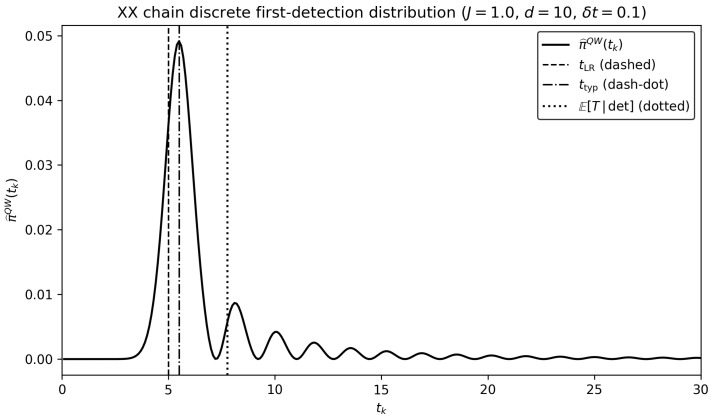
Discrete stroboscopic information arrival distribution in an XX spin chain. First-detection probability mass function ${\hat{\pi }}^{QW}\left(t_{k}\right)$ for J=1, d=10, and temporal resolution δt=0.1. The dashed line indicates the Lieb–Robinson time $t_{\mathrm{LR}}=d/\left(2J\right)$, the dash–dotted line marks the typical arrival time $t_{\mathrm{typ}}$ (mode), and the dotted line marks the conditional mean E[T|det]. The plot is restricted to t<30 to emphasize the ballistic peak and early time suppression.

Figure 4 shows the discrete first-detection distribution ${\hat{\pi }}^{QW}\left(t_{k}\right)$ associated with the stroboscopic protocol. Importantly, ${\hat{\pi }}^{QW}\left(t_{k}\right)$ depends on the experimental resolution δt, here taken to be δt=0.1. For $t_{k}<t_{\mathrm{LR}}=d/\left(2J\right)$, the detection probabilities $p_{\det}\left(t_{k}\right)$ are strongly suppressed, consistent with the Lieb–Robinson exponential attenuation. The distribution exhibits a peak near $t_{\mathrm{typ}}\approx d/\left(2J\right)$, reflecting propagation at the maximal group velocity $v_{\max}=2J$ in the one-excitation sector. The right-skewed tail originates from the combination of residual late-time detection probabilities and the discrete survival factor $S_{k}=\prod_{j<k}\left(1-p_{\det}\left(t_{j}\right)\right)$, which enforces the requirement of no earlier detection. The conditional mean arrival time is computed directly from the discrete mass function as $E\left[T|\det\right]=\left(\sum_{k}t_{k}\;{\hat{\pi }}^{QW}\left(t_{k}\right)\right)/\left(\sum_{k}{\hat{\pi }}^{QW}\left(t_{k}\right)\right)$, and typically lies to the right of the mode due to the tail. We emphasize that E[T∣det] is an expected arrival time conditional on detection, since the total detection probability $P_{\det}=\sum_{k}{\hat{\pi }}^{QW}\left(t_{k}\right)$ need not equal unity under the discrete stroboscopic protocol and the unconditional expectation would diverge whenever $P_{\det}<1$. Indeed, the total detection probability $P_{\det}=\sum_{k}{\hat{\pi }}^{QW}\left(t_{k}\right)$ need not equal unity under the stroboscopic protocol, since detection is only attempted at discrete sampling times.

Table 1 displays the Lieb–Robinson arrival time $t_{\mathrm{LR}}=d/\left(2J\right)$, the typical arrival time $t_{\mathrm{typ}}$ (mode of the QW distribution), the expected arrival time E[T∣det] (conditional on detection) and the total detection probability $P_{\det}$, for several values of the coupling *J* and distance *d*. We observe that all characteristic times scale approximately linearly with the distance *d*, as expected from ballistic propagation in the one-excitation sector. For each set of parameters, the discrete first-detection distribution satisfies

$$t_{\mathrm{LR}}<t_{\mathrm{typ}}<E\left[T|\det\right],$$

where $t_{\mathrm{typ}}$ denotes the mode of the discrete mass function ${\hat{\pi }}^{QW}\left(t_{k}\right)$ and E[T∣det] is the conditional mean arrival time computed from the discrete distribution. We find that the conditional mean arrival time can significantly exceed the Lieb–Robinson time $t_{\mathrm{LR}}=d/\left(2J\right)$ for certain parameter values, while the discrete first-detection distribution ${\hat{\pi }}^{QW}\left(t_{k}\right)$ does not vanish for $t_{k}<t_{\mathrm{LR}}$, the associated probabilities are exponentially suppressed, reflecting the light-cone structure encoded in the Lieb–Robinson bound. Consequently, identifying $t_{\mathrm{LR}}$ with a characteristic arrival time may lead to a substantial underestimation of the expected latency. In this sense, the waiting time distribution provides a more complete information-theoretic characterization of propagation times beyond the envelope constraint imposed by Lieb–Robinson bounds.

## 5. Discussion, Conclusions and Outlook

This paper presents an analysis of three relevant notions of time-of-arrival distributions for a discrete quantum system that correspond to fundamentally different questions. The QC distribution is a conditional distribution of time given that the system is in the arrival state. The QF distribution is an unconditional distribution describing the rate at which probability flows into that state. The QW distribution is a waiting time distribution describing the time until the first observed arrival along many independent identical systems. These notions are very general and can be extended to discrete open systems that interact with their environment and obey non-unitary dynamics governed by completely positive trace-preserving (CPTP) maps or, in the Markovian limit, by a Lindblad master equation. In these more general settings, *transition probabilities* $p_{i\to j}\left(t\right)$ remain well-defined as experimentally accessible occupation statistics under a single-measurement (stroboscopic) protocol. It is important to emphasize again that these distributions are not alternative proposals for the same observable, but should be regarded as answers to distinct operational questions. This distinction is especially relevant in quantum information science, where different tasks such as circuit execution times, error detection latencies, or communication and synchronization protocols, may naturally call for different notions of arrival time, while the present work is primarily foundational, the framework developed here suggests several concrete domains in which operational notions of time-of-arrival (TOA) distributions may play a substantive role in quantum technologies, and we leave for further research the development of such applications in realistic open-systems and many-body settings.

A first natural domain is quantum sensing and threshold detection under false alarm constraints. In realistic sensing tasks, decisions are made under competing hypotheses ($H_{1}$ signal present versus $H_{0}$ background only), and performance is intrinsically latency-constrained. In contrast to approaches based solely on instantaneous occupation probabilities or steady-state error rates, one can perform optimization as a functional problem over a suitable TOA distribution to maximize the early time probability detection for a given confidence level expressed in terms of premature clicks. This single-measurement perspective would complement the analysis of continuous quantum measurement and quantum trajectories [13,21], where first-click statistics are experimentally accessible and central to quantum detection theory [22].

A second potential domain of application is in quantum computing, particularly in qubit reset scheduling and tail risk control. Modern quantum processors operate under stringent cycle-time budgets, and reset protocols must guarantee readiness within prescribed latency windows. Experimental demonstrations of measurement-based and active reset already emphasize the importance of stochastic readiness times [23,24]. In this setting, suitably defined TOA distributions can be used to quantify both the expected reset time and the probability of deadline violation, providing distributional information beyond mean occupation dynamics. A closely related application concerns information propagation in locally interacting quantum systems. As discussed in the XX spin chain toy model, Lieb–Robinson bounds rigorously constrain the maximal velocity of information spreading [16,25], but they provide worst-case envelopes rather than operational access times. TOA distributions associated with specific detection protocols could instead characterize when excitations or logical information become experimentally accessible at a remote site. In this sense, TOA concepts complement velocity bounds by introducing a probabilistic notion of information arrival time, which may help enhance available estimates based on circuit depth time performance in many body systems.

Finally, many quantum communication protocols, including photon-based transmission, entanglement swapping, and teleportation, are fundamentally conditioned on successful events such as photodetection or Bell-state measurement outcomes. The timing of such events is central to synchronization, feed-forward operations, and optimization in quantum networks [26,27,28]. A relevant notion of TOA consistent with the measurement protocol in place could therefore provide a natural framework to analyze conditional success versus latency trade-offs in distributed quantum architectures. Taken together, these examples suggest that TOA distributions can prove to be powerful operational tools for latency-aware quantum control, detection, and communication protocols.

More generally, beyond technological applications, the present analysis contributes to the conceptual problem of time in quantum mechanics. The absence of a self-adjoint time operator canonically conjugate to the Hamiltonian [1] implies that time-of-arrival cannot be treated on the same footing as position or momentum observables. Our results show explicitly that distinct operational definitions of arrival time naturally give rise to different distributions, even within the standard unitary formalism of quantum mechanics. In this sense, the “time-of-arrival problem” does not admit a unique resolution at the level of formalism alone, but must be understood relative to a specified measurement protocol. This plurality of operational definition of quantum time is consistent with earlier analyses of arrival versus detection times [18,29,30,31], and with relational or conditional approaches to time [4,32]. Rather than competing answers to a single question, the QF, QC, and QW constructions clarify that different notions of time correspond to different experimentally meaningful questions. In this respect, this work supports the view that time in quantum mechanics is not a primitive observable, but an operationally emergent concept defined through measurement context.

## Figures and Tables

**Table 1:** Discrete-time comparison of Lieb–Robinson time, typical arrival time, and conditional mean arrival time for the XX chain under the stroboscopic first-detection protocol. Sampling times are $t_{k}=k\;\delta t$ with δt=0.1. The first-detection mass function is ${\hat{\pi }}_{d}^{QW}\left(t_{k}\right)=\left(\prod_{j=1}^{k-1}\left(1-p_{\det}\left(t_{j}\right)\right)\right)p_{\det}\left(t_{k}\right)$ with $p_{\det}\left(t_{k}\right)=J_{d}{\left(2Jt_{k}\right)}^{2}$. The reported mean is $E\left[T|\det\right]=\left(\sum_{k}t_{k}\;{\hat{\pi }}^{QW}\left(t_{k}\right)\right)/\left(\sum_{k}{\hat{\pi }}^{QW}\left(t_{k}\right)\right)$ and $P_{\det}=\sum_{k}{\hat{\pi }}^{QW}\left(t_{k}\right)$. Here, $v_{\mathrm{LR}}=2J$.

*J*	*d*	$t_{\mathrm{LR}}=\frac{d}{2J}$	$t_{\mathrm{typ}}$	E[T∣det]	$P_{\det}$
0.50	5	5.000	5.200	5.387	1.000
0.50	10	10.000	10.500	10.930	1.000
0.50	20	20.000	20.900	22.020	1.000
1.00	5	2.500	2.800	3.873	1.000
1.00	10	5.000	5.500	7.764	1.000
1.00	20	10.000	10.700	15.353	0.999
2.00	5	1.250	1.500	6.625	0.995
2.00	10	2.500	2.800	11.294	0.990
2.00	20	5.000	5.500	18.891	0.983

## Data Availability

The original contributions presented in this study are included in the article. Further inquiries can be directed to the corresponding author.
